# The role of the thalamus in modular functional networks in temporal lobe epilepsy with cognitive impairment

**DOI:** 10.1111/cns.14345

**Published:** 2023-07-09

**Authors:** Xiaomin Pang, Xiulin Liang, Weiwei Chang, Zongxia Lv, Jingyuan Zhao, Peirong Wu, Xinrong Li, Wutong Wei, Jinou Zheng

**Affiliations:** ^1^ Department of Neurology Guangxi Medical University First Affiliated Hospital Nanning China

**Keywords:** cognitive impairment, functional magnetic resonance imaging, modular network, temporal lobe epilepsy, thalamus

## Abstract

**Objective:**

Cognitive deficit is common in patients with temporal lobe epilepsy (TLE). Here, we aimed to investigate the modular architecture of functional networks associated with distinct cognitive states in TLE patients together with the role of the thalamus in modular networks.

**Methods:**

Resting‐state functional magnetic resonance imaging scans were acquired from 53 TLE patients and 37 matched healthy controls. All patients received the Montreal Cognitive Assessment test and accordingly were divided into TLE patients with normal cognition (TLE‐CN, *n* = 35) and TLE patients with cognitive impairment (TLE‐CI, *n* = 18) groups. The modular properties of functional networks were calculated and compared including global modularity Q, modular segregation index, intramodular connections, and intermodular connections. Thalamic subdivisions corresponding to the modular networks were generated by applying a ‘winner‐take‐all’ strategy before analyzing the modular properties (participation coefficient and within‐module degree z‐score) of each thalamic subdivision to assess the contribution of the thalamus to modular functional networks. Relationships between network properties and cognitive performance were then further explored.

**Results:**

Both TLE‐CN and TLE‐CI patients showed lower global modularity, as well as lower modular segregation index values for the ventral attention network and the default mode network. However, different patterns of intramodular and intermodular connections existed for different cognitive states. In addition, both TLE‐CN and TLE‐CI patients exhibited anomalous modular properties of functional thalamic subdivisions, with TLE‐CI patients presenting a broader range of abnormalities. Cognitive performance in TLE‐CI patients was not related to the modular properties of functional network but rather to the modular properties of functional thalamic subdivisions.

**Conclusions:**

The thalamus plays a prominent role in modular networks and potentially represents a key neural mechanism underlying cognitive impairment in TLE.

## Graphical abstract

The functional thalamic subdivisions participated in interactions among multiple modular functional networks in TLE patients. The integrative functions of thalamus in the reorganization of the modular network might be a key neural mechanism underlying cognitive impairment in TLE patients.

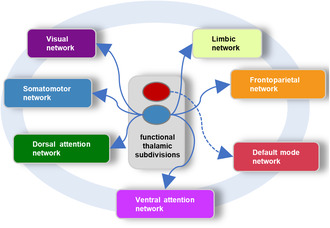

## 1 INTRODUCTION

Temporal lobe epilepsy (TLE) is classified as localization‐related symptomatic epilepsy with seizure activity originating from a hippocampal, amygdala, parahippocampal focus, or lateral temporal lobe neocortex.^1^, ^2^, ^3^ Large‐scale epidemiological studies are scarce and difficult as diagnosis requires appropriate clinical seizure semiology, electroencephalographic features and neuroimaging for confirmation.^4^ But there is no doubt that TLE is the most common type of localization‐related epilepsy diagnosed in tertiary care centers.^5^ In addition to recurrent spontaneous seizures, patients with TLE often suffer from cognitive impairment. Up to 50%–80% of patients with TLE demonstrate impairment in at least one cognitive domain, most frequently in memory.^6^ Other aspects of cognitive functioning have also been reported to be affected, including attention, executive function, judgment, insight, and problem solving.^7^, ^8^, ^9^ These widespread cognitive consequences may cause a significant decline in the quality of life for TLE patients and are sometimes more debilitating than the seizures themselves.^10^ Although different studies have provided important information on vulnerability factors for cognitive deficits in TLE patients,^11^, ^12^ the neurobiological mechanisms underlying cognitive impairment remain unclear.

Over the past decades, accumulating evidence from semeiology, histopathology, electrophysiology, and neuroimaging have revealed that the neurobiological insults in TLE extend beyond the epileptogenic temporal lobe, supporting the concept that TLE is a network disorder rather than a focal disease process.^13^, ^14^ The introduction of graph theory, a mathematical framework for quantifying the topological architectures of complex network systems, has provided novel and valuable insights into the pathophysiology of cognitive burden in TLE.^15^ In our previous work, we applied graph theory approach to functional magnetic resonance imaging (fMRI) data to identify associations between topological properties of large‐scale networks and cognitive performances in TLE. We demonstrated that the functional network in TLE patients with impaired alertness tended towards randomization, showing less small‐worldness and less normalized clustering.^16^ Variance in global network efficiency was also associated with worse cognitive performance in language and conceptual thinking.^17^ Global network measures, such as global efficiency, clustering coefficient, and shortest path length, as well as regional centrality measures for regional nodes, have been confirmed to effectively capture the features behind the cognitive dysfunction in TLE and to predict the severity of cognitive comorbidities in a study conducted by Mazrooyisebdani et al.^18^ Struck et al.^19^ also demonstrated a closer relationship between the global breakdown of the typical modular network connectivity of the brain and worse neuropsychological phenotypes in TLE.

Although cognitive impairment in TLE is increasingly recognized to result from network disorganization, most studies omit the contribution of the thalamus to the ensemble networks.^20^, ^21^ As a heterogeneous subcortical structure, the thalamus projects a wide interconnection to the cerebral cortex.^22^ Such anatomical features are ideal for mediating information transfer across multiple cortical regions.^22^, ^23^ Previous studies have used fMRI to investigate the functional anatomy of the thalamus, revealing that it can be parcellated into various functional thalamic subdivisions based on its functional connectivity with cortical regions.^24^, ^25^, ^26^, ^27^ A recent graph‐theoretic analysis by Hwang et al.^28^ further revealed that these functional thalamic subdivisions make distinct contributions to the organization of brain network. The brain network follows a modular architecture that allows rapid and efficient sharing of information between brain regions.^29^, ^30^, ^31^, ^32^ Within the modular networks, the functional thalamic subdivisions can act as provincial hubs to support communication within a particular cortical functional network, or as connector hubs to mediate interactions across multiple cortical functional networks.^28^ Moreover, the authors also found that focal thalamic lesions lead to a disruption of the modular structure of the cortical functional network, further highlighting the critical contribution of thalamic function to brain network organization.^28^ There is no doubt that the thalamus is an integrative hub in the brain network.

On the other hand, thalamic abnormalities in TLE are already well‐known from imaging studies.^26^, ^33^, ^34^, ^35^ Several studies have also reported a significant relationship between thalamic abnormalities and cognitive impairment in TLE. For instance, Seidenberg et al.^36^ found that hippocampal volumes in TLE patients showed a selective association with verbal memory performance, while thalamic volume was significantly correlated with measures of both memory and non‐memory function, suggesting that thalamic integrity plays a key role in characterizing the nature and extent of the cognitive impairment observed in TLE. In the study by Zhang et al., ^37^ the structural and functional connectivity of the left dorsal lateral prefrontal cortex–thalamic pathway was significantly increased in TLE patients with executive control deficits compared to the patients with normal executive control and healthy controls. In our previous work, disruption of the thalamocortical system in TLE patients was correlated with alertness performance.^38^ Further investigation of the role of the thalamus in modular networks may provide important evidence for understanding the mechanisms of cognitive impairment in TLE. To date, a comprehensive understanding of the contribution of the thalamus to modular networks in TLE patients is still lacking.

Thus, we collected resting‐state fMRI data from patients with TLE and systematically investigated the modular architecture of the functional networks associated with distinct cognitive states. To assess the thalamic contribution to the modular organization of functional networks, we generated thalamic subdivisions corresponding to modular networks and then analyzed the modular properties of each subdivision. The relationship between modular properties and cognitive performance was further explored. We entertain the hypothesis that the thalamus is a critical hub that disrupts the modular organization of the functional network, resulting in extensive cognitive impairment in TLE.

## 2 MATERIALS AND METHODS

### 2.1 Participants

Fifty‐three patients with TLE were consecutively enrolled through the epilepsy clinic of the First Affiliated Hospital of Guangxi Medical University from December 2018 to February 2020. Diagnosis of TLE was made by at least two experienced neurologists based on comprehensive clinical evaluation, including history, seizure semiology, physical and neurological exams, ictal/interictal scalp electroencephalogram recordings and regular structural MRI according to the epilepsy classification of the International League Against Epilepsy (ILAE).^39^ Patient inclusion criteria involved age from 18 to 60 years, right‐handedness, existing characteristic features of unilateral TLE, and no cortical malformations or other focal lesions found on structural MRI. Patients were excluded if any other mental or neurological disorders were known including a history of traumatic brain injury or encephalitis, poor cooperation with experimental procedures, contraindication to MRI, and history of substance or alcohol abuse. During the same recruitment interval, 37 healthy volunteers without a history of mental or neurological diseases were recruited from community sources as the healthy control (HC) group. Controls were matched to the patient groups with respect to age, gender, handedness, and years of education. Demographic data are shown in Table 1. This study was approved by the Medical Research Ethics Committee of the First Affiliated Hospital of Guangxi Medical University, and written informed consent was obtained from each subject.

**TABLE 1:** Demographic characteristics and clinical features of patients with TLE and HC.

	TLE‐CI (*n* = 18)	TLE‐CN (*n* = 35)	TLE whole sample (*n* = 53)	HC (*n* = 37)	Statistical value	*p* Value
Demographic characteristics						
Age (years)	33.39 ± 9.47	29.49 ± 7.92	30.81 ± 8.59	29.57 ± 7.75	1.607^a^	0.206^b^
					0.703^c^	0.484^d^
Sex (male/female)	6/12	8/27	14/39	16/21	3.364^a^	0.186^e^
					2.777^c^	0.096^e^
Education level (years)	12 (12–15)	15 (12–15)	15 (12–15)	15 (12–15)	0.964^a^	0.618^f^
					−0.265^c^	0.791^g^
Handedness (R/L)	18/0	35/0	53/0	37/0	NA	NA
Clinical features						
Age at onset (years)	25.61 ± 10.32	19.03 ± 9.40	21.26 ± 10.12	NA	2.336	0.023^d^
Epilepsy duration (years)	6 (3.5–9.375)	10.5 (5.5–14)	7.5 (5.25–13.25)	NA	−1.835	0.067^g^
Seizure frequency (seizures per month)	0.5 (0–4.125)	1 (0–3)	1 (0–3)	NA	−0.602	0.547^g^
AEDs (mono−/polytherapy)	10/8	16/19	26/27	NA	0.227	0.634^e^
MoCA scores	23.11 ± 2.78	28.14 ± 1.19	26.43 ± 3.04	28.84 ± 1.34	75.762^a^	<0.001^b^
					−4.505^c^	<0.001^d^

*Note*: For all tests, *p* < 0.05 was considered statistically significant.

Abbreviations: HC, healthy control; TLE‐CI, TLE with cognitive impairment; TLE‐CN, TLE with cognitive normal.

^a^ Test stats and statistical value among the three group comparisons.

^b^ *p* Value was obtained by one‐way ANOVA.

^c^ Test stats and statistical value of the TLE whole sample and HC comparisons.

^d^ *p* Value was obtained by a two‐sample *t*‐test.

^e^ *p* Value was obtained by the chi‐square test.

^f^ *p* Value was obtained by a Kruskal–Wallis *H*‐test.

^g^ *p* Value was obtained by a Mann–Whitney *U*‐test.

### 2.2 Cognition assessment

All participants were administered brief cognitive screening in the form of the Montreal Cognitive Assessment (MoCA) tool.^40^ Previous studies have suggested that MoCA can be used as an appropriate screening tool for cognitive impairment in epilepsy patients, as it showed better sensitivity and specificity than other brief instruments such as Mini‐Mental (MMSE).^41^, ^42^ We utilized the Chinese version of MoCA.^43^ According to the suggested cut‐off score, the patients were classified as TLE with cognitive impairment (TLE‐CI) if their scores were <26; all other patients were classified as TLE with normal cognition (TLE‐CN).^43^

### 2.3 MRI data acquisition

MRI data were acquired from all participants using an Achieva 3.0 T MRI system scanner (Philips) with a standard eight‐channel head coil. Functional MRI data were acquired using an echo‐planar imaging sequence (repetition time: 2000 ms, echo time: 30 ms, flip angle: 90°, field of view: 220 × 220 mm, matrix: 64 × 62 mm, 31 transverse slices, slice thickness: 3.5 mm with interslice gap: 0.5 mm, resulting in 3.4 × 3.4 × 4.0 mm^3^ voxels). During scanning, the participants were instructed to remain in an awake and relaxed state, keeping their eyes closed and avoiding thinking about any specific topic. 3D T1‐weighted MRI data were acquired using the turbo field echo sequence (repetition time: 7.8 ms, echo time: 3.4 ms, flip angle: 9°, field of view: 256 × 256 mm, matrix size: 256 × 256 mm, 176 sagittal slices, slice thickness: 1 mm with no gap, resulting in 1.0 × 1.0 × 1.0 mm^3^ voxels).

### 2.4 Imaging data preprocessing

Preprocessing of functional data was performed using the Data Processing and Analysis for Brain Imaging toolbox (DPABI V4.2, www.rfmri.org/dpabi), which is based on Statistical Parametric Mapping software (SPM12, www.fil.ion.ucl.ac.uk/spm) on the Matrix Laboratory (MATLAB R2013b, https://www.mathworks.com/) platform. The first 10 volumes were removed to allow for signal stabilization. The remaining volumes underwent slice timing correction and realignment. Considering that functional connectivity analysis is sensitive to head motion effects, subjects were excluded when head motion exceeded 3 mm in displacement or 3° in angular rotation, or if their mean framewise displacement values were greater than 0.2 mm.^44^ The individual functional images were warped to a standard space by applying the transformation matrix derived from registering the T1 image (co‐registered with mean functional images) into the Montreal Neurological Institute (MNI) template using unified segmentation, and thereafter resampled to a resolution of 3 × 3 × 3 mm^3^. Linear trends were removed from the time courses and temporal band‐pass filtering was performed (0.01–0.08 Hz). The images were then spatially smoothed with a 6 mm full width at half‐maximum Gaussian kernel. Finally, Friston‐24 head motion parameters,^45^, ^46^ the averaged white matter signal and the cerebrospinal fluid signal were regressed out as confounding variables.^47^ Given that the thalamus is a relatively small structure, we did not perform any spatial smoothing in the thalamic functional parcellation to avoid signal blurring.

### 2.5 Modular analysis of cortical–cortical functional network

Following preprocessing, the construction of cortical–cortical functional network was performed with the graph theoretical network analysis toolbox (GRETNA v2.0, www.nitrc.org/projects/gretna). An undirected graph was formed for each participant, with nodes defined as 200 cortical regions as reported by Schaefer et al.,^48^ and edges assessed by computing Pearson's correlation coefficients between all pairs of cortical regions, resulting in a 200 × 200 correlation matrix. For each individual cortical–cortical functional network, the global modularity Q, the modular segregation index (MSI), intramodular connections, and intermodular connections were calculated.^49^ Seven referenced modular subnetworks were generated according to a previous report by Yeo et al.,^50^ i.e., visual network (VIS), somatomotor network (SMN), dorsal attention network (DAN), ventral attention network (VAN), limbic network (LIM), frontoparietal network (FPN), and DMN. To ensure our results were not biased by a single specific threshold, all graph metrics were calculated across a range of thresholds (the sparsity threshold ranged from 0.1 to 1 with an interval of 0.1), which is widely used in graph theory‐based network studies.^51^, ^52^, ^53^, ^54^ We reported results of the area under the curve (AUC) for each topological property across thresholds.

### 2.6 Functional parcellation of the thalamus

Different thalamic subdivisions have distinct functional connectivity with the cortex. We, therefore, generated a functional parcellation by applying a ‘winner‐take‐all’ strategy to identify functional boundaries in the thalamus. The procedure basically followed the steps described in the classic articles by Zhang et al.^25^ and Fair et al.^55^ Briefly, voxel‐wise functional connectivity analysis was performed using the seven defined modular subnetworks ROIs mentioned above as seeds. The mean functional connectivity values restricted to the Harvard‐Oxford thalamus probabilistic atlas (threshold at 10%) across all participants were extracted. The strength of functional connectivity for seven modular subnetworks ROIs within each thalamic voxel was then compared. The ‘winner‐take‐all’ represents labeling each thalamic voxel to the modular subnetwork with the strongest functional connectivity values. The resulting thalamic subdivisions were used for subsequent ROI‐wise functional connectivity and nodal properties analysis.

### 2.7 Thalamic nodal properties of thalamocortical functional networks

To test our hypothesis that the thalamus plays a critical role in the modular structure of the brain network, we investigated the participation coefficient (PC) and within‐module degree z‐score (WMD) of each thalamic subdivision refer to the previous study by Hwang et al^28^ Briefly, the thalamocortical functional network was constructed for each participant, with nodes for 200 cortical regions defined by Schaefer et al.,^48^ and seven thalamic functional subdivisions, and edges were assessed by computing Pearson's correlation coefficients between all pairs of regions, resulting in a 207 × 207 correlation matrix. To ensure that our results were not biased by a single specific threshold, the graph metrics, i.e., PC and WMD, were calculated across a range of thresholds (the sparsity threshold ranged from 0.1 to 1 with an interval of 0.1). We reported the results of AUC for each topological property across thresholds. Note that thresholding the entire thalamocortical functional network would likely sacrifice thalamic–cortical connections, and we therefore applied a two‐step thresholding approach. In brief, thresholds were individually applied to the cortical–cortical connectivity matrices (matrix size, 200 × 200), thalamic–thalamic connectivity matrices (matrix size, 7 × 7), and thalamic–cortical connectivity matrices (matrix size, 7 × 200) before combining into the thalamocortical functional network.

### 2.8 Statistical analysis

Statistical analyses of the demographics, clinical characteristics, and modular measures were conducted using Statistical Package for the Social Sciences (SPSS V 22.0, www.ibm.com/spss). A normal distribution test was applied to all quantitative data using the one‐sample Kolmogorov–Smirnov test. To determine the significance of the group differences for normally distributed data, independent‐samples *t*‐test or one way ANOVA was used, while the Mann–Whitney *U*‐test or Kruskal–Wallis *H*‐test was used for non‐normally distributed data. A chi‐square test was performed to assess the differences in categorical data. Correlation analyses were conducted to detect associations between MoCA performance scores and altered modular properties. The significance criterion was *p* < 0.05, and the Bonferroni correction was applied for multiple comparisons.

## 3 RESULTS

### 3.1 Demographics, clinical characteristics, and cognitive performance

Fifty‐three patients with TLE and 37 matched HC were included in the study. No overall significant differences were found in age, sex, and education level between the TLE and HC groups (*p* > 0.05). According to MoCA scores, 18 (34%) patients with TLE were classified as TLE‐CI, while 35 (66%) were TLE‐CN. Among TLE‐CI and TLE‐CN and HC groups, no significant differences were observed with regard to age, sex, and education level (*p* > 0.05). In terms of clinical characteristics, the duration of epilepsy, seizure frequency, and the number of antiepileptic drugs (AEDs) currently taken did not differ between the TLE‐CI and TLE‐CN groups (*p* > 0.05). But notably age of epilepsy onset in the TLE‐CN groups was earlier than for the TLE‐CI group (*p* < 0.05). All demographic data and clinical features are summarized in Table 1.

### 3.2 Modular properties of cortical–cortical functional network

**FIGURE 1:**
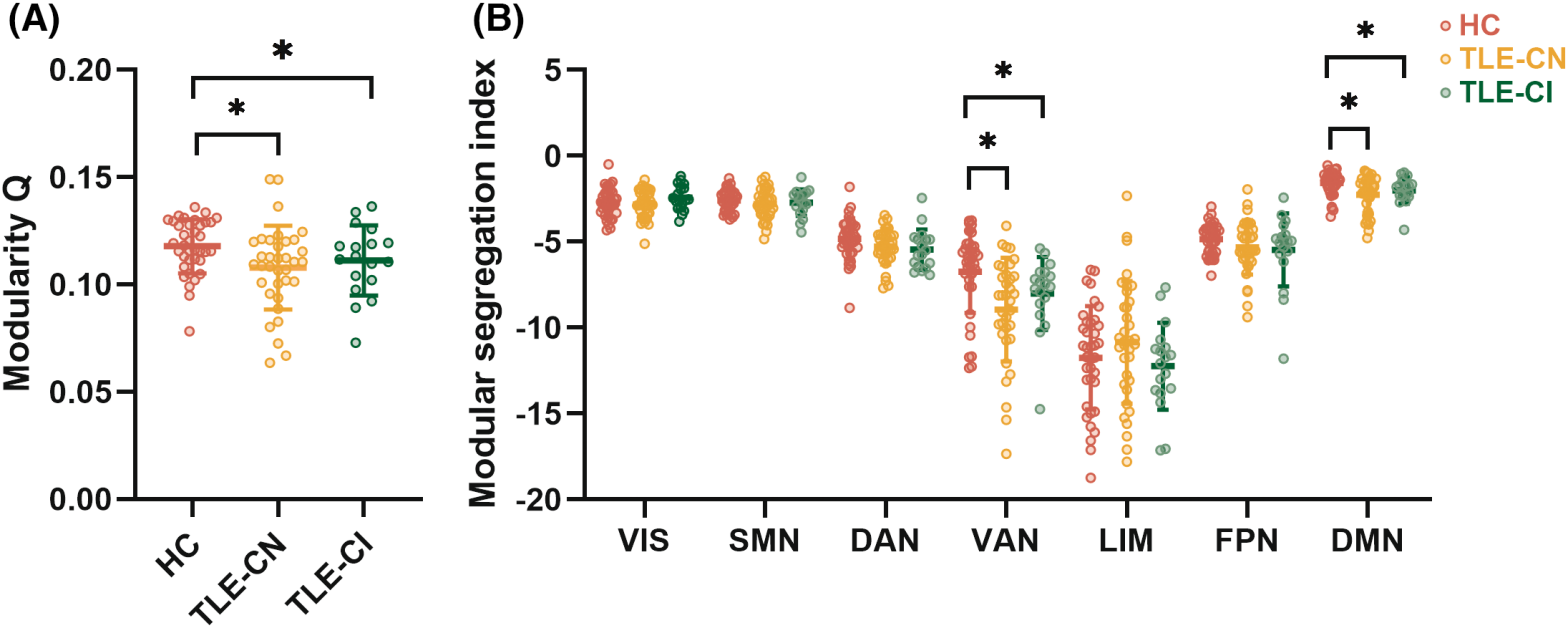
Module analysis in the cortical network. (A) Group comparison in modularity among the three groups. The horizontal lines above the dot plots indicate comparisons achieving statistically significant thresholds (*p < 0.05, Bonferroni corrected). (B) Group comparison in modular segregation indices among the three groups. The horizontal lines above the dot plots indicate comparisons that achieved statistically significant thresholds (*p < 0.05, Bonferroni corrected). HC, healthy controls; TLE‐CN, TLE with cognitive normal; TLE‐CI, TLE with cognitive impairment.

**FIGURE 2:**
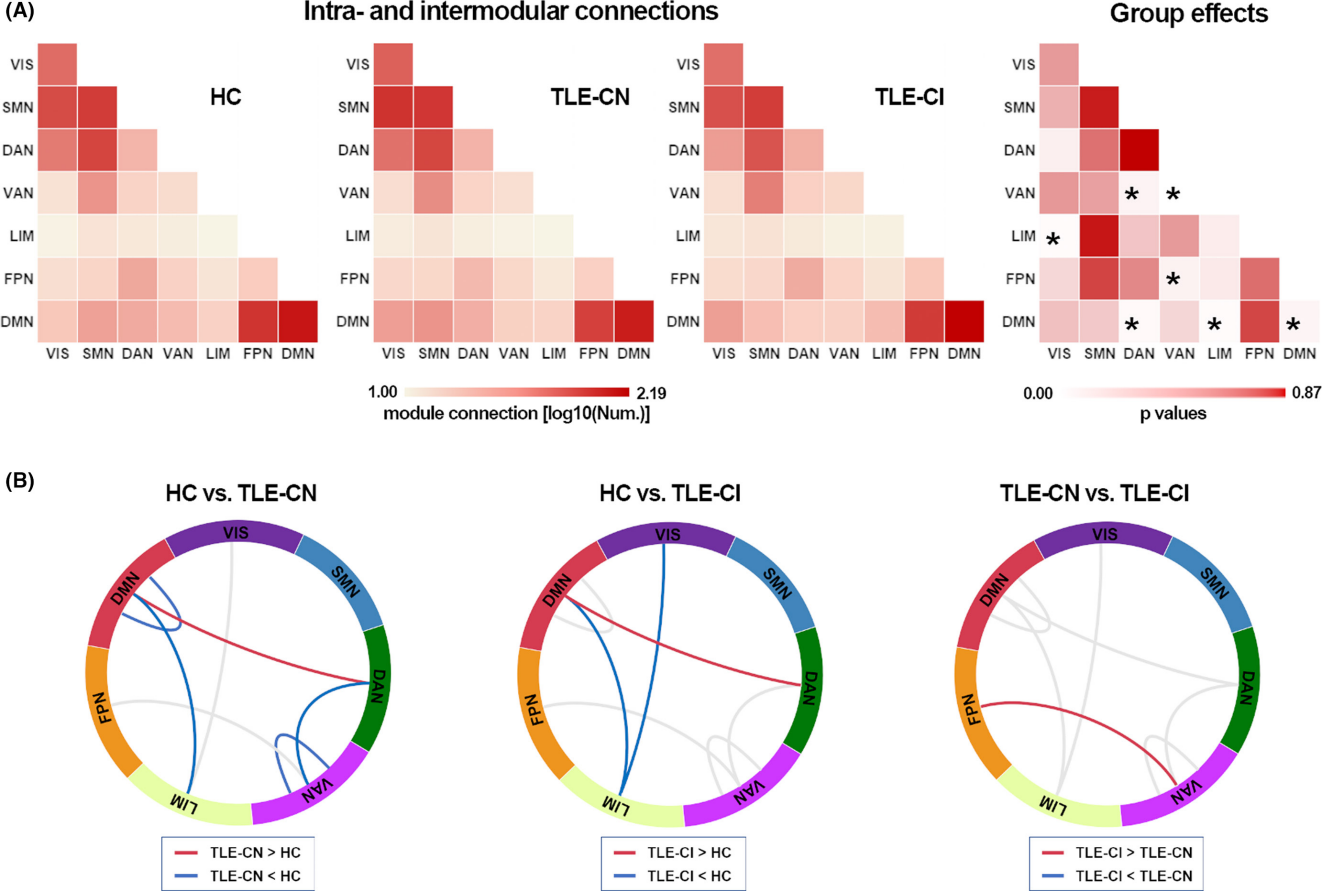
The intra‐ and intermodular connections among the three groups. (A) The mean matrices of intra‐ and intermodular connection in three groups and the group effects are presented. The symbol ‘*’ indicates the connectivity variability that demonstrated significant group effects (*p < 0.05, Bonferroni corrected). (B) Between‐group differences in intra‐ and intermodular connections for each pair of groups. Red and blue lines indicate significantly more connections and fewer connections, respectively.

In the cortical–cortical functional network, modularity Q showed significant group differences (*p* < 0.05). Both TLE‐CN and TLE‐CI groups displayed significantly lower modularity compared to the HC group (Figure 1A). Moreover, there were also decreases in MSI values in the VAN and DMN in both TLE‐CN and TLE‐CI groups (*p* < 0.05; Figure 1B). Further analyses revealed significant group effects on the intramodular connections within VAN and DMN, and intermodular connections, including VIS‐LIM, DAN‐VAN, DAN‐DMN, VAN‐FPN, and LIM‐DMN (*p* < 0.05). The TLE‐CN group exhibited a greater number of DAN‐DMN intermodular connections, but fewer DAN‐VAN and LIM‐DMN intermodular connections as well as intramodular connections within VAN and DMN compared with the HC group. The TLE‐CI group exhibited a greater number of DAN‐DMN intermodular connections, but fewer VIS‐LIM and LIM‐DMN intermodular connections than the HC group. The TLE‐CI group exhibited a greater number of VAN‐FPN intermodular connections than the TLE‐CN group (Figure 2).

### 3.3 Parcellation of the thalamus and pattern of thalamocortical connections

**FIGURE 3:**
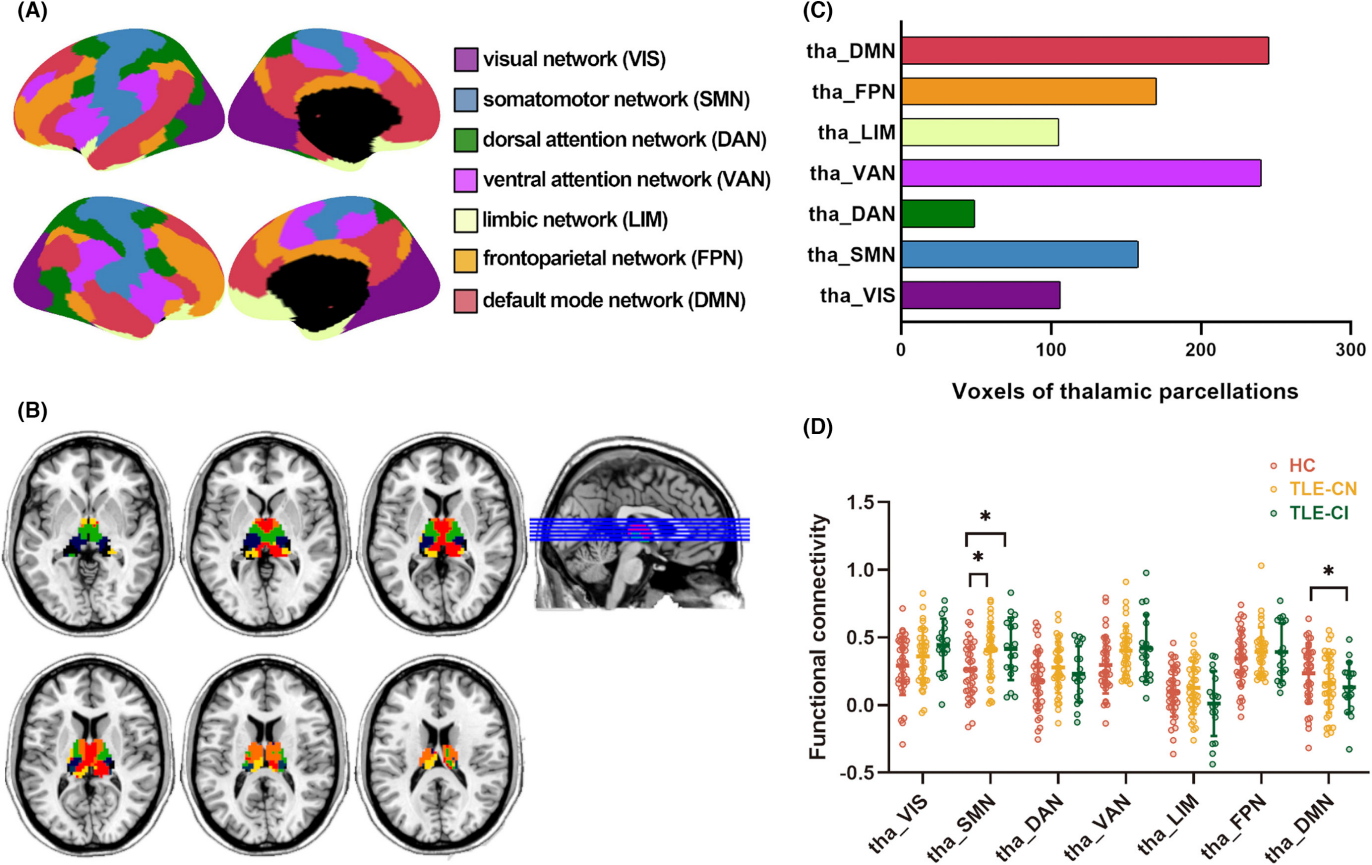
Thalamic parcellations based on functional connectivity. (A) Seven cortical seeds were selected according to the classical functional network, i.e., visual network, somatomotor network, dorsal attention network, ventral attention network, limbic network, frontoparietal network, default network (provided by Professor Alexander Schaefer). (B) A “winner takes all” approach was adopted to parcel and color code the thalamus at voxel level. Thalamic parcellations were termed as tha_VIS, tha_SMN, tha_DAN, tha_VAN, tha_LIM, tha_FPN and tha_DMN. Color codes are in concordance with those represented the cortical seeds shown in (A). (C) The distribution of voxels in each thalamic subdivision. (D) Functional connectivity between each thalamus subregion and the corresponding functional network. The horizontal lines above the dot plots indicate comparisons that achieved statistically significant thresholds (*p < 0.05, Bonferroni corrected).

The bilateral thalamus was parcellated into seven functional subdivisions (Figure 3A,B). The subdivisions were designated as the visual network (tha_VIS), somatomotor network (tha_SMN), dorsal attention network (tha_DAN), ventral attention network (tha_VAN), limbic network (tha_LIM), frontoparietal network (tha_FPN), and default mode network (tha_DMN). The voxel distribution of each thalamic functional subdivision is shown in Figure 3C, and differences among groups in the functional connectivity between thalamic functional subdivision and the corresponding functional network are presented in Figure 3D. Compared with HC group, both TLE‐CN and TLE‐CI groups displayed significantly increased connectivity in the thalamic–SMN network (*p* < 0.05), whereas decreased connectivity in the thalamic–DMN network was only observed in the TLE‐CI group (*p* < 0.05).

### 3.4 Thalamic nodal properties of thalamocortical functional connectivity

**FIGURE 4:**
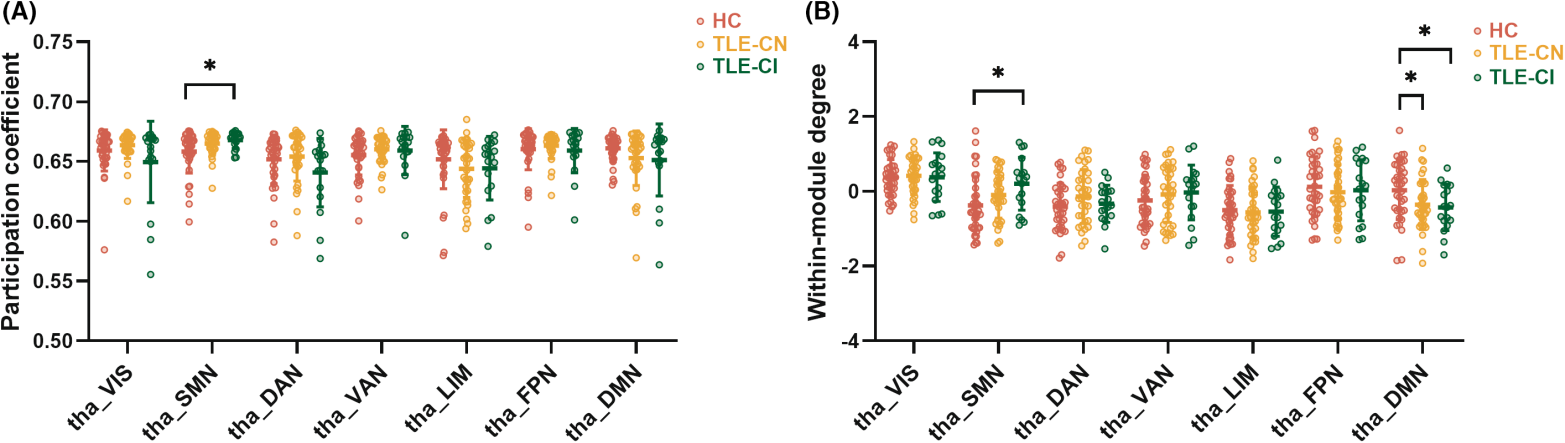
Group differences in thalamocortical participation coefficients (A) and within‐module degree (B). The horizontal lines above the dot plots indicate comparisons that achieved statistically significant thresholds (*p < 0.05, Bonferroni corrected).

To determine the thalamic contribution to modular organization of functional network, we assessed the PC and WMD of each thalamic functional subdivision. We observed significant group differences involving PC in the tha_SMN subdivision (*p* < 0.05; Figure 4A). Compared to the HC group, the TLE‐CI group showed higher PC in the tha_SMN subdivision, whereas the TLE‐CN group showed no significant differences. Furthermore, significant group differences in WMD were found in the tha_SMN and tha_DMN subdivisions (*p* < 0.05; Figure 4B). Compared to the HC group, the TLE‐CI group showed higher WMD in the tha_SMN subdivision, but for the TLE‐CN group, there were no significant differences. Both the TLE‐CN and TLE‐CI groups showed lower WMD in the tha_DMN subdivision compared to the HC group.

### 3.5 Correlation between network properties and MoCA scores

**FIGURE 5:**
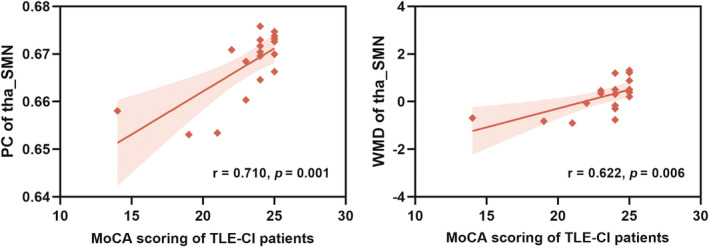
Correlation analysis of modular properties between with cognitive scores in TLE‐CI patients.

Correlation analyses were performed to test the relationship between the abnormalities in modular properties and the decline of cognitive performance in TLE. As seen in Figure 5, the PC and WMD values of the tha_SMN were positively correlated with MoCA scores in TLE‐CI patients (*p* < 0.05). There were no correlations between other modular properties and MoCA scores in TLE‐CI patients (*p* > 0.05). And similarly, no correlations were found between all modular properties and MoCA scores in TLE‐CN patients (*p* > 0.05).

## 4 DISCUSSION

Cognitive deficits are common in patients with TLE.^7^ In this study, we focused on the modular architecture of functional networks associated with distinct cognitive states in TLE patients and the role of the thalamus in a modular network. Several notable findings emerged: (1) both TLE‐CN and TLE‐CI patients exhibited lower global modularity, as well as lower modular specialization in VAN and DMN; nevertheless, different intramodular and intermodular connection patterns related to cognitive states were observed. (2) The functional thalamic subdivisions in TLE presented different hub properties in the modular network. The tha_DMN showed lower WMD values in both TLE‐CN and TLE‐CI groups, while the tha_SMN exhibited higher PC and WMD values in TLE‐CI group. (3) The cognitive performance in TLE patients was not correlated with modular properties of functional networks but correlated with the abnormal thalamic hub properties. The present findings suggested a prominent role for the thalamus in information interactions of the modular networks, proposing this as a key neural mechanism underlying cognitive impairment in TLE patients.

The modular organization of brain networks facilitates the rapid specialization and integration of information in response to cognitive processes.^31^, ^56^ The study by Hermann et al.^57^ presented distinct modular feature patterns of structural covariance networks associated with the cognitive phenotypes in TLE. Each patient group (generalized cognitive impairment, focal cognitive impairment, and no cognitive impairment groups) showed a significant difference relative to HC for modularity Q.^57^ In a white matter network study, the language and memory impaired group of TLE patients also showed decreased modularity with respect to HC across a consecutive range of network densities.^58^ Most of the existing research has focused on the structural network, as it is considered that abnormal structural network properties are more closely related to cognitive phenotypes than the disordered functional network properties in the pathology of epilepsy.^59^ Here, we investigate the global modularity Q values of the functional network associated with the distinct cognitive states of TLE patients. We found that both the TLE‐CN and TLE‐CI groups showed significantly lower modularity Q compared to the HC group, suggesting a decreased functional integration of network organization in TLE patients. However, global modularity analysis did not reveal significant differences between the TLE‐CN and TLE‐CI patients. In addition, the modularity index was not associated with cognitive performance. We speculate that the reduced network integration results from the functional network reorganization in the epileptic brain, but this does not fully explain the broad cognitive impairment seen in TLE patients.

The decreased modularity observed could be caused by reductions in within‐network connectivity or increased between‐network connectivity. We, therefore, further quantified the intramodular and intermodular connections. Between the seven defined modular subnetworks, the DMN is described as the neurologic basis for the self and plays an important role in advanced human thought processes.^60^ Disruptions of the DMN in TLE patients have been extensively investigated, for example, an early report showed decreases in cerebral blood flow in regions belonging to the DMN during seizure activity in TLE.^61^ Moreover, the significant deactivation of DMN brain regions occurred simultaneously with the appearance of interictal epileptic discharges in TLE patients.^62^ The decreased functional connectivity within the DMN was also described in TLE.^63^ These decreases in connectivity reflected the functional impairments associated with the duration of epilepsy and may be a consequence of the reduced connection density underpinning structural connectivity degeneration.^63^, ^64^ Attention is another important cognitive function. The VAN reflects bottom‐up stimulus‐driven attention corresponding to the infrequent or unexpected events that are behaviorally relevant.^65^ Disrupted VAN in TLE patients was thought to be associated with the functional impairment in attention.^66^ In agreement with previous studies, we observed fewer intramodular connections within the DMN and VAN in the TLE‐CN group compared to HC. Why no differences were evident in the TLE‐CI group remains an intriguing question, but we suggest that TLE‐CI patients may over‐recruit brain regions within the DMN and VAN to meet functional demands. Thus, stronger compensation in the connectivity would result in the absence of anomalous intramodular connectivity in the TLE‐CI group. Nevertheless, changes in intramodular and intermodular connections still lead to a decrease in MSI in both VAN and DMN in the TLE‐CN and TLE‐CI groups, indicative of worsened functional segregation in these two networks in TLE patients.

In addition to the changes in the intramodular connections, our results show different intermodular connection patterns between groups. Both the TLE‐CN and TLE‐CI groups showed significantly greater DAN‐DMN connections compared to the HC group. Unlike the internally directed processes in the DMN, DAN reflects external‐oriented cognitive processing.^67^ The interaction between these two networks flexibly reconfigures across different cognitive states, offering the opportunity for information exchange that allows perception to inform internally oriented thinking, and the converse is also true.^68^ Amer et al.^69^ investigated age differences in the interaction between the DAN and DMN, reporting that a greater DMN‐DAN anticorrelation was associated with better performance in distraction regulation in older adults. Therefore, the DAN‐DMN interaction pattern was considered a compensating mechanism. In support, mild cognitive impairment (MCI) showed enhanced anticorrelation between the DMN and DAN compared to healthy elderly subjects, and a stronger DMN‐DAN anticorrelation was associated with better cognitive performance.^70^ However, in Alzheimer's disease (AD), the anticorrelation strength dropped to nearly zero.^70^ A possible explanation for this phenomenon was that the dysfunctional interactions between DMN and DAN eventually overcome the compensatory mechanism as MCI progresses to AD.^70^ We speculate that the greater DAN‐DMN intermodular connections observed here in both the TLE‐CN and TLE‐CI groups also reveal adaptation and functional compensation in brain networks. This compensatory reorganization of the brain network helps to maintain cognition and behavior at a relatively normal level and avoids more severe cognitive dementia and behavioral abnormalities. DMN alterations in TLE patients have been proposed to be based on the rich connections between the hippocampus and the key regions within the DMN.^71^ Consistent with previous studies showing abnormal network patterns in TLE, we found there were fewer LIM‐DMN intermodular connections in both TLE‐CN and TLE‐CI groups. Moreover, when we compared the connectivity patterns of TLE‐CN and TLE‐CI patients, only differences in VAN‐FPN were found. The greater VAN‐FPN intermodular connections in the TLE‐CI group compared to the TLE‐CN group may also reflect a compensatory enhancement in response to the loss of network function, or alternatively may result from less efficient neural processing in the TLE as concluded in a recent graph theory analysis.^20^ Interestingly, these features of reduced network separation and modular connection patterns did not show a significant correlation with cognitive performance in our study.

Higher‐order cognition is a cortical–centric process in the classical view, and the contribution of the thalamus to cognition is not well recognized.^72^ In fact, as a heterogeneous subcortical structure, thalamic nuclei have distinct inputs and outputs. The first‐order thalamic nuclei receive inputs from peripheral sensory organs or other subcortical structures and send projections to primary sensory and motor cortical areas; the high‐order thalamic nuclei receive inputs predominantly from the cortex, which projects back to the cerebral cortex.^73^ The extensive interconnections between the cerebral cortex and thalamus highlight its role in corticocortical communication. The lateral geniculate nucleus modulates thalamocortical transmission and plays an important role in controlling attentional response gain and visual awareness far beyond the visual cortex gateway.^74^ The mediodorsal thalamus also amplifies prefrontal cortical connectivity to sustain attentional control without relaying categorical information.^75^ Clearly, the thalamus acts not only as a passive relay but also as an active gatekeeper to control the information interactions across cortical networks. Given this central role in cortical functioning, the involvement of the thalamus in cognitive processes has received increasing attention over the past two decades. It has been found that focal thalamic lesions, such as thalamic infarction in humans, may lead to specific cognitive and behavioral symptoms, including impaired episodic memory, executive dysfunction, and attention deficits.^76^ Thalamic abnormalities have also been confirmed in patients with clinical manifestations of cognitive impairment such as AD and Parkinson's disease.^77^, ^78^ In view of the presence of thalamic pathologies found in TLE,^30^, ^31^, ^32^ it is necessary to explore the role played by the thalamus in cognitive deficits. To date, studies have reported a link between local thalamic atrophy and cognitive decline.^36^, ^79^ Several studies have reported a significant relationship between thalamic–hippocampal or thalamic–prefrontal cortex connectivity and cognitive performance in TLE.^37^, ^80^, ^81^ We, therefore, focused specifically on the role of the thalamus in the modular networks in this study.

Based on the strongest functional connectivity to the cortical functional networks, the current study identified seven thalamic subdivisions using a ‘winner‐take‐all’ approach. The thalamic subdivisions, namely, tha_VIS, tha_SMN, tha_DAN, tha_VAN, tha_LIM, tha_FPN, and tha_DMN, corresponded to the VIS, SMN, DAN, VAN, LIM, FPN, and DMN modular networks and thus represented distinct functional features. Of these thalamic subdivisions, tha_DMN had the largest number of voxels, indicating the dominant functional coupling to the DMN in the thalamus. Perhaps, a more interesting point was that all subdivisions showed near hemispheric symmetry. Thus, it appears that thalamic function might not exhibit a pronounced lateralization effect. In the ROI‐wise functional connectivity analysis, we demonstrated abnormality of thalamocortical functional connectivity. Compared to HC, the thalamic–SMN connectivity was increased in both TLE‐CN and TLE‐CI groups, while the thalamic‐DMN connectivity decreased in the TLE‐CI group. Together, these changes in thalamocortical connectivity patterns highlight the central role of the tha_SMN and tha_DMN in TLE patients.

To better understand the topological properties of the thalamus, we further calculated the WMD and PC for each thalamic subdivision. The WMD and PC represent measures of intra‐ and inter‐modular connectivity density for nodes in the network, respectively. As an integrative hub in the brain network that supports modality selective and multimodal integrative processes, the thalamus should exhibit high WMD and PC.^28^ Indeed, we observed that the tha_DMN showed lower WMD values in both the TLE‐CN and TLE‐CI groups, indicating that the thalamus exhibits less connectivity to cortical regions belonging to the DMN network. The weakened ‘provincial hub’ properties of the tha_DMN in TLE patients suggests that the thalamus cannot effectively support information communication towards the execution of specialized functions in the DMN network. This observation also seems capable of explaining the worse functional segregation of the DMN in TLE patients.

In addition, the tha_SMN exhibited higher PC and WMD values in the TLE‐CI group, providing an indication that the thalamus exhibited stronger connections with cortical regions in the corresponding SMN network, as well as in the other multiple networks. The stronger ‘provincial hub’ and ‘connector hub’ properties of the tha_SMN suggest that the thalamus facilitated intranetwork communication in the SMN, as well as inter‐network communication. Given that the brain is a remarkably adaptive and plastic organ,^82^ we infer that the enhanced hub property of the tha_SMN may reflect restructuring in the epileptic brain. This compensation in TLE‐CI patients is necessary to protect the epileptic brain from more severe cognitive dementia and behavioral abnormalities. Indeed, this assumption was confirmed by the results of correlation analyses. The WMD and PC values of the tha_SMN were positively correlated with MoCA scores in TLE‐CI patients, indicating a relationship between the enhancement in ‘provincial hub’ and ‘connector hub’ properties and better cognitive performance.

Taken together, we show those functional thalamic subdivisions participate in multiple modular functional network interactions in TLE patients. The decline in cognitive performance in TLE patients was associated with abnormal thalamic hub properties. These findings highlight that the integrative function of the thalamus in TLE may be a key neural mechanism underlying cognitive impairment in TLE patients. Notably, brain stimulation techniques targeting the anterior nucleus of the thalamus have now been approved for the treatment of medically refractory epilepsy and have helped reduce seizures.^83^ There is also evidence that deep brain stimulation may lead to improvements in attention and executive function.^84^ Our findings here on the role of the thalamus in modular networks may provide a new theoretical basis for brain stimulation therapy in TLE.

Finally, the limitations of our study need to be considered. First, the profile of cognitive dysfunction in TLE patients exhibits substantial variability. Notably, cognitive performance in TLE ranges from intact to significantly impaired in different functional domains, including language, memory, attention, executive, and visuospatial functions. We also only performed a group‐level analysis in TLE patients with distinct cognitive states according to the MoCA cognitive screening tool. Given the heterogeneity of cognitive profiles in TLE, a more comprehensive battery of neuropsychological tests would better help identify abnormalities associated with specific cognitive phenotypes. Second, all patients included in the study were treated with AEDs. This poses the challenge of dissociating the relative contribution of AEDs from the disease‐specific observations reported in this study. Finally, long‐term follow‐up studies are necessary, which would provide an essential step towards a better understanding of the thalamocortical network abnormalities associated with cognitive trajectories.

## 5 CONCLUSIONS

Our study demonstrated that functional thalamic subdivisions are involved in the reorganization of the modular networks in TLE patients, suggesting that it is intimately involved in the mechanism of cognitive impairment. The findings presented extend our understanding of the contribution of the thalamus to cognitive impairment in TLE and may guide the development of new treatment strategies.

## CONFLICT OF INTEREST STATEMENT

All authors claim that there are no conflicts of interest.

## Data Availability

The data that support the findings of this study are available from the corresponding author upon reasonable request.
